# Cation/Anion Substitution in Cu_2_ZnSnS_4_ for Improved Photovoltaic Performance

**DOI:** 10.1038/srep35369

**Published:** 2016-10-17

**Authors:** Balakrishna Ananthoju, Jeotikanta Mohapatra, Manoj K. Jangid, D. Bahadur, N. V. Medhekar, M. Aslam

**Affiliations:** 1 IITB-Monash Research Academy, IIT Bombay, Powai, Mumbai, 400076, India; 2 Department of Physics, IIT Bombay, Powai, Mumbai, 400076, India; 3 Centre for Research in Nanotechnology and Science (CRNTS), IIT Bombay, Powai, Mumbai, 400076, India; 4 Department of Metallurgical Engineering and Materials Science, IIT Bombay, Powai, Mumbai, 400076, India; 5 Department of Materials Engineering, Monash University, Clayton, VIC-3800, Australia; 6 National Centre for photovoltaic Research and Education, IIT Bombay, Powai, Mumbai, 400076, India

## Abstract

Cations and anions are replaced with Fe, Mn, and Se in CZTS in order to control the formations of the secondary phase, the band gap, and the micro structure of Cu_2_ZnSnS_4_. We demonstrate a simplified synthesis strategy for a range of quaternary chalcogenide nanoparticles such as Cu_2_ZnSnS_4_ (CZTS), Cu_2_FeSnS_4_ (CFTS), Cu_2_MnSnS_4_ (CMTS), Cu_2_ZnSnSe_4_ (CZTSe), and Cu_2_ZnSn(S_0.5_Se_0.5_)_4_ (CZTSSe) by thermolysis of metal chloride precursors using long chain amine molecules. It is observed that the crystal structure, band gap and micro structure of the CZTS thin films are affected by the substitution of anion/cations. Moreover, secondary phases are not observed and grain sizes are enhanced significantly with selenium doping (grain size ~1 μm). The earth-abundant Cu_2_MSnS_4_/Se_4_ (M = Zn, Mn and Fe) nanoparticles have band gaps in the range of 1.04–1.51 eV with high optical-absorption coefficients (~10^4^ cm^−1^) in the visible region. The power conversion efficiency of a CZTS solar cell is enhanced significantly, from 0.4% to 7.4% with selenium doping, within an active area of 1.1 ± 0.1 cm^2^. The observed changes in the device performance parameters might be ascribed to the variation of optical band gap and microstructure of the thin films. The performance of the device is at par with sputtered fabricated films, at similar scales.

Quaternary chalcogenides of the I_2_–II–IV–VI_4_ group have attracted extensive attention due to their exceptional optoelectronic device characteristics^1^,^2^,^3^,^4^,^5^. One of the most important applications of quaternary chalcogenides is their utility as light absorbing materials in the harvesting of solar energy, which is possible because of their high absorption coefficients (α ~ 10^4 ^cm^−1^), suitable optical band gap (1.4–1.6 eV), low-cost production, and low toxicity^6^,^7^. A well-known quaternary chalcogenide that is used in commercial thin film solar cells is CIGS (CuInGaSe_2_). However, limited availability of In and Ga seriously limits their application in the mass production of solar cells^8^. On the other hand, inexpensive, non-toxic and earth-abundant photovoltaic materials are of significant interest. In recent times, Cu_2_ZnSnS_4_ (CZTS) has emerged as an excellent prospect for photovoltaic devices due to its good absorption coefficient, earth abundance (of Cu, Zn, Sn and S), and a suitable band gap (1.5 eV)^9^,^10^,^11^,^12^. Solar cells fabricated with CZTS have shown lower efficiencies when compared with CIGS solar cells (≈23%)^13^,^14^. Furthermore, CZTS has a band gap of 1.5 eV, and it has achieved an efficiency of 8.4% thus far, which is still lesser than the efficiency of CZTSSe (≈12.6%)^3^,^15^. Recently, there have been attempts to enhance the efficiency of CZTSSe solar cells by altering the S/Se ratio to get the optimum band gap and appropriate film microstructure^16^. However, precise control of the S/Se ratio is very difficult during the thermal annealing process. Moreover, achieving an appropriate crystal phase and controlled stoichiometry during synthesis is still a big challenge^17^. Furthermore, the formation of non-stoichiometric compositions (secondary phases) such as Cu_2_SnS_3_, Cu_2_S, SnS_2_ and ZnS is often observed along with the CZTS phase^17^,^18^. Thus far, it has been understood that these secondary phases suppress the performance of the device by acting as recombination centers^18^. Therefore, altering the metal ions in CZTS might be a good alternative to control the band gap and the microstructure.

The incorporation of extrinsic impurities such as Fe and Mn (to replace Zn) or Se (to replace S) in the wurtizite structure of Cu_2_ZnSnS_4_ offers better flexibility and control over the stoichiometry and the crystal phase^19^. The cationic substitution in Cu_2_MSnS_4_/Se_4_ (M = Zn, Mn and Fe) helps in tuning the optoelectronic properties of the quaternary chalcogenides. Typically it may lead to an improvement in the band gap energy from 1.0 eV to 1.5 eV and also suppress the formation of the secondary phase^19^. To date, there are only a few reports on the fabrication of Cu_2_MSnS_4_/Se_4_ (M = Zn, Mn and Fe) nanoparticles based devices^20^,^21^,^22^,^23^,^24^,^25^,^26^. Very recently, J. Chu *et al*. reported the first CFTS solar cell with 0.07% power conversion efficiency^27^, wherein, they found that a thickness of 600 nm as absorber might not be sufficient to absorb the light. In addition, Wong and co-workers also demonstrated the fabrication of ‘9% efficiency’ cation substituted CZTS solar cell with Cd substitution^28^, wherein, cadmium, a toxic element could be doped up to 4 mol% with an efficiency enhancement factor of two in comparison to pristine CZTS^28^. Also, Chu *et al*. reported the CMTS solar cells with 0.49% power conversion efficiency^29^. The poor efficiency of CMTS (M = Mn) solar cells is attributed to the high interface resistance between CMTS and CdS layers^29^. As per our knowledge, these are the only few reports on the substitution of anion and cations in CZTS. Therefore, further research is essential to understand the influence of anion/cation substitution on the structural, optical and electrical properties of the absorber films in order to achieve high efficiency solar cells. Sol-gel, hot-injection, spray pyrolysis, solvothermal and hydrothermal synthesis methods have been adopted to synthesize Cu_2_MSnS_4_/Se_4_ (M = Zn, Fe, and Mn) nanoparticles^20^,^21^,^22^,^23^,^24^,^25^,^26^,^30^. The solution process offers a convenient method for the formation of quaternary chalcogenide nanoparticles that have the desired stoichiometry and phase. This technique enables a cheaper method for the fabrication of low-cost photovoltaic films when compared to vacuum-based techniques. Unfortunately, most of the nanoparticle-synthesis approaches discussed thus far involve complex chemistry (solvent and two or more reducing or oxidizing agents and organic metal precursors) and expensive precursors to render sub-gram quantities of mono-disperse nanoparticles^20^,^21^,^22^,^23^,^24^,^25^,^26^. In all these attempts to synthesize the nanoparticles, issues such as phase purity, stoichiometry, and uniformity of the quaternary chalcogenide nanoparticles are not addressed systematically. Furthermore, from the industry perspective, a simple, economic and scalable approach needs to be designed to fabricate mono-disperse nanoparticles and hence their ink.

In this article, we report a simple and economic approach to synthesize quaternary chalcogenide (Cu_2_MSnS_4_/Se_4_, M = Zn, Mn and Fe) nanoparticles. We studied the influence of the incorporation of extrinsic impurities such as Fe and Mn (to replace Zn) or Se (to replace S) on the optical, electrical transport and photovoltaic device-performance characteristics of nanoparticle thin films. We demonstrate the synthesis of as much as 20 g of quaternary chalcogenide nanoparticle powder in a single reaction, without a size-sorting process. Furthermore, a scalable, binder-free ink was prepared, with toluene as the dispersion solvent. These inks were coated on Mo-coated glass substrates by a simple knife-coating method. Multiple heat treatments were performed in order to remove surfactants from the surface of the nanoparticles and improve the conductivity of the films. We investigate the influence of ionic substitution on the structural, optical and electrical properties of the CZTS thin films. In addition to the basic characterizations on absorber layers, we fabricate thin film solar cells and evaluated their solar cell performance.

## Experimental Section

### Materials

Copper chloride (CuCl_2_), zinc chloride (ZnCl_2_), stannous chloride (SnCl_2_), manganese chloride (MnCl_2_), iron chloride (FeCl_2_), sulfur powder, selenium powder, oleylamine, acetone, hexane, and toluene were used in the synthesis of quaternary chalcogenide nanoparticles. All chemicals were purchased from Sigma-Aldrich and were used without any further purification.

## Experimental Methods

The as-prepared nanoparticles were characterized using XpertPANAlytic X–ray diffractometer with Cu–K_α_ radiation (λ = 1.54 Å), field emission gun scanning electron microscope (FEGSEM) (JEOL JSM-7600F FEG-Scanning Electron Microscope) operated at 15 kV, energy-dispersive X-ray analysis (EDX), inductively coupled plasma atomic emission spectroscopy (ICP-AES) characterization techniques and field emission gun transmission electron microscopy (FEGTEM) images were obtained with JEOL JEM 2100F at an accelerating voltage of 200 kV. The optical properties of quaternary chalcogenide nanoparticles were measured by UV-visible absorption spectroscopy (Lambda 950-Perkin Elmer). The electrical properties (carrier concentration, Hall mobility, and electrical resistivity) were acquired using a Hall Effect measurement system (Model 8404 AC/DC, Lake Shore) with an applied magnetic field intensity of 0.50 T by a four-point probe using Van der Pauw geometry. I–V curves of the thin films measured using a solar simulator (AM 1.5G, 100 mW/cm^2^). The photo voltaic (PV) characteristics of the fabricated devices were measured using a Keithley 2400 source meter and a Newport solar simulator (model number 91160) with simulated AM 1.5G solar illumination. External quantum efficiency (EQE) measurements were carried out by a double source illumination system (Xenon lamp, QTH lamp). All measurements were performed at room temperature.

### Gram-scale synthesis of CMTS/Se (M = Zn, Mn and Fe) nanoparticles

**Figure 1:**
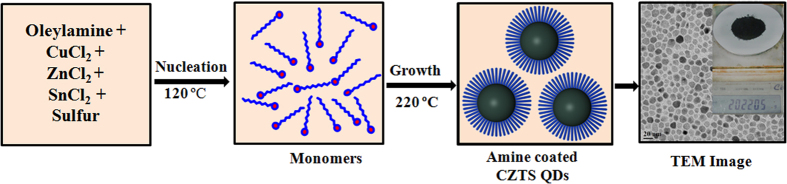
Schematic flow diagram of amine functionalized CZTS nanoparticle synthesis. As indicated, after nucleation at 120 °C, the solution color changes from blue to brownish, which indicates the formation of the metal-amine complex; growth of CZTS at 220 °C leads to a blackish solution due to the nucleation formation of CZTS nanoparticles. The growth process is complete approximately in about 0.5 hrs.

First, we describe a simple one-step approach to synthesize 20.2 g of Cu_2_ZnSnS_4_ nanoparticle powder from metal chloride precursors using a long-chain amine as the solvent, reducing and surface-functionalizing agent. In a typical synthesis of 6 nm-sized Cu_2_ZnSnS_4_ nanoparticles, 50 mM of CuCl_2_, 25 mM ZnCl_2_, and 25 mM SnCl_2_ were added to 600 mM of oleylamine (amine-to-precursor molar ratio of 1:6) in a three-neck round-bottomed flask. The reaction mixture was heated at 120 °C for 15 min. to form the metal-amine complex under a nitrogen atmosphere. At this point, 100 mM of sulfur was added into the mixture, resulting in an immediate change in the color of the reaction mixture to light brown. Later, the temperature was raised to 220 °C at a rate of 5 °C/min, with further heating for 2 h. The obtained nanoparticles were collected from the growth solution by precipitation with acetone, followed by centrifugation and, hence, re-dispersion in hexane. After several hexane/acetone extractions, the final product was dried under vacuum. A brief pictorial detail of the synthesis procedure is shown as a schematic view (Fig. 1). The same synthesis formulation is also extended to synthesize of Cu_2_ZnSnSe_4_, Cu_2_ZnSn(S_0.5_Se_0.5_)_4_, Cu_2_MnSnS_4_, and Cu_2_FeSnS_4_ nanoparticles.

### Preparation of nanoparticle ink and thin film deposition

As-prepared nanoparticles were dispersed in toluene (200 mg/mL) to make nanoparticle ink. The dispersed nanoparticles were ball milled for 10 h to formulate a uniform ink. The toluene-based ink was found to be stable for many months without precipitation. These inks were deposited onto various substrates (fluorine-doped tin oxide (FTO), soda-lime glass, and molybdenum (Mo-coated glass) by simple knife-coating (also known as doctor blade method)^15^. Typically, a small amount of the concentrated nanoparticle ink is drop-casted on the edge of a substrate; adhesive tape is then used as a doctor-blading guide. The thickness of the tape determines the initial thickness of the doctor-bladed film. A circular glass rod was used to deposit the films. A second coat of the ink was then applied in a manner similar to the first one to fill the cracks in the first layer, resulting in the final film. Each layer of the nanoparticle films was found to be ~(0.4 ± 0.1) μm in thickness as measured by SEM cross sectional analysis. The substrate was immediately coated with the CZTS layer, after substrate was covered with a narrow tube end funnel to prevent fast evaporation of the solvent. Fast evaporation of solvent could create cracks in the film, which is undesirable. After each coating, the thin films were annealed at 400 °C (heating rate 3 °C/min) for 30 min with a continuous flow of argon gas in a tubular furnace to remove the capping agent (amine in our case) and to improve the conductivity. Note that the low heating rate (3 °C/min) is used to avoid crack formation in the films. After annealing, the films were sulfurized (or selenized) using sulfur (or selenium) along with tin powder (in a separate boat) at 550 °C for 30 minutes with a continuous flow of argon gas in a tubular furnace in order to get larger grain size and single phase Cu_2_MSnS_4_/Se_4_ (M = Zn, Mn and Fe) form. The residual sulfur was removed by rinsing the film in isopropyl alcohol for 2 min. Figure S1 shows the as-deposited and post-heat-treated thin films of all the samples that were deposited over the soda lime and Mo-coated glasses. The modified solution process is cheaper and renders an improved, denser, and more continuous film.

### Device fabrication

The device stack consisted of glass/Mo/absorber (800–1000 nm)/CdS (50–60 nm)/i-ZnO (110 nm)/Al: ZnO (300 nm)/Ni/Al (contact) (Fig. S2). A 50-nm thick CdS film (which acts as a buffer layer) was deposited by chemical bath deposition using a CdSO_4_, ammonia, and (NH_2_)_2_SC aqueous solution at 80 °C. Furthermore, intrinsic ZnO (acts as an n-type layer) was deposited on CdS by dielectric sputtering, and Al-doped ZnO (which acts as a window layer) was deposited on intrinsic ZnO by metal sputtering. At the top of the layered deposited surface, Al contacts were deposited using the thermal evaporation technique. Finally, the total area (1.1 ± 0.1 cm^2^) of all the devices was defined by mechanical scribing of the samples.

## Results and Discussion

**Figure 2:**
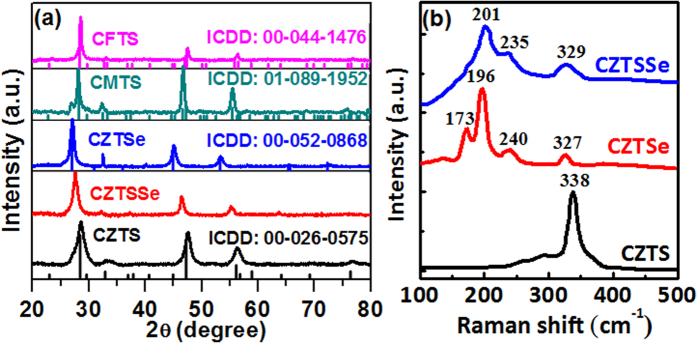
The powder XRD patterns of kesterite and zincblende Cu_2_MSnS_4_/Se_4_ (M = Zn, Mn and Fe) nanoparticles. The substitution of different metals leads to change in lattice volume as indicated by the peak shift toward the lower diffraction angle, (**b**) Raman spectra of CZTS, CZTSe, and CZTSSe sulfurized/selenized nanoparticles thin film samples.

X-ray diffraction (XRD) measurements were carried with all nanoparticles in order to analyze the crystal phase. Figure 2 shows the XRD patterns (along with corresponding ICDD patterns) of Cu_2_ZnSnS_4_, Cu_2_FeSnS_4_, Cu_2_MnSnS_4,_ Cu_2_ZnSnSe_4_, Cu_2_ZnSn(S_0.5_Se_0.5_)_4_ nanoparticles that were prepared at a large-scale. The CZTS sample show major peaks at 28.5, 47.3, and 56.1, 2θ-values, which can be indexed as (112), (220) and (312) planes, corresponding to the kesterite structure (ICDD No: 00-026-0575). The strongest intensity of the (112) peak suggests the favored orientation effect on the structure of the crystal. When there is partial substitution of sulfur with Se atoms the XRD peaks shift to lower 2θ angles. After complete replacement of sulfur with Se, we obtain CZTSe, the XRD peaks of which are appear at lower 2θ angles when compared to both CZTS and CZTSSe. The shifting of the XRD peaks to lower angles implies an increase in lattice parameter values, which is expected, given the larger atomic radius of Se (atomic radius ~ 0.198 nm) in comparison to sulfur (atomic radius ~ 0.184 nm)^31^. When Zn is substituted with Mn, the nanoparticles show a zincblende crystal structure, which matches quite well with the reported literature^22^. CFTS diffraction patterns can be indexed to the zincblende structure, which corresponds similar with the reported literature^32^. The lattice parameters are calculated for all the nanoparticles from the XRD data and presented in Table 1. We found that there is an increase in the value of the lattice parameters when partial or total replacement of sulfur with selenium is achieved. According to Salome *et al*., the (112) peak position of CZTSSe is used to estimate the ratio of S/(S + Se)^33^. The (112) peak position (2θ = 27.55°) of CZTSSe lies between the CZTS and CZTSe. The relation between the (112) peak position and the ratio is expressed by the following equation^33^:


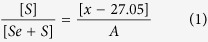


where *A* is a linear factor and takes the value 1.45, which results from the (112) peak position difference between the CZTS and CZTSe, and *x* is the CZTSSe peak position. By using this equation the composition ratio is estimated to be 34%, which is close to the EDS results.

The XRD results of the ternary and quaternary nanoparticles alone cannot confirm their crystal structure; this is because of the same peak positions for two phases (kesterite and stannite) as well as for secondary phases such as ZnS and Cu_2_SnS_3_^34^. Therefore, we applied Raman spectroscopy measurements for the phase analysis of all sulfurized/selenized nanoparticle thin films (Fig. 2b). The most intense Raman peak in CZTS is observed at 338 cm^−1^, which corresponds to the A_1_ phonon mode of the kesterite structure of CZTS and agrees well with the literature^35^. The major Raman peak of CZTSe is observed at 196 cm^−1^, which also corresponds to the A_1_ phonon mode of the kesterite phase^36^. The shift of the A_1_ vibration mode to lower energies is due to the substitution of S by Se. The CZTSSe shows the peak at 201 cm^−1^, which is located in between the CZTS and CZTSe A_1_ vibration mode^37^. The mid-location of the CZTSSe peak is ascribed to the change in the lattice vibrations after incorporation of S into the CZTSe lattice structure^38^. The peak shift is consistent with the XRD results. For the CFTS sample, Raman peaks are observed at 285 and 319 cm^−1^ (Fig. S3a). The de-convoluted peaks at 285 cm^−1^ and 319 cm^−1^ can be attributed to Cu_2_FeSnS_4_^39^,^40^ (Fig. S3b). The major peak at 319 cm^−1^ is due to the strongest asymmetrical vibration of a pure anion mode of the sulfur atom around the Sn metal^39^,^40^. The peak at 285 cm^−1^ corresponds to a pure anion mode around the Cu cation^39^,^40^. The CMTS also shows (Fig. S3a) a strong peak positioned at 328 cm^−1^, which is again attributed to the A_1_ mode of CMTS^29^. Thus, the XRD and Raman results confirm that our nanoparticles are in requisite phase and secondary phases are not present in as-synthesized samples.

The as-synthesized nanoparticles are capped by the long chain amine groups and organic solvents (toluene) used in the ink formulation also contain carbon atoms. However, organic impurities need to be removed from the films for realistic applications^41^. If a solvent is not removed completely, it will leave carbon residues on the surface of the as-synthesized nanoparticle film. The carbon residues can reduce the surface energy of the particles and hinder the growth of nanoparticle thin films^41^. Thus, for the grain growth to occur, the surface of the nanoparticles needs to be cleaned properly. At high-temperature heat treatment, the solvent evaporates and thus it gets removed from the film. In order to analyze the carbon residues in the as-deposited, annealed and sulfurized CZTS thin films, Raman spectroscopy (Fig. S4) measurements are performed to estimate the carbon presence in the CZTS films qualitatively. The Raman measurements show the peaks positioned at 1350 and 1582 cm^−1^ in the as-deposited films, which correspond to D and G peaks of graphitic carbon^42^. The intensities of D and G peaks were drastically suppressed after annealing the films at 400 °C in an argon gas atmosphere. After sulfurization, the D and G peaks completely disappear; this indicates that the carbon residues are completely removed from the films. The complete removal of carbon residues might be due to interaction of carbon with sulfur vapor to form CS_2_, COS, and H_2_S^43^. Moreover, if rich carbon residues are present in the sample, then more sulfur is required to interact with these carbon residues so as to form a clean surface for the crystal growth^43^.

**Figure 3:**
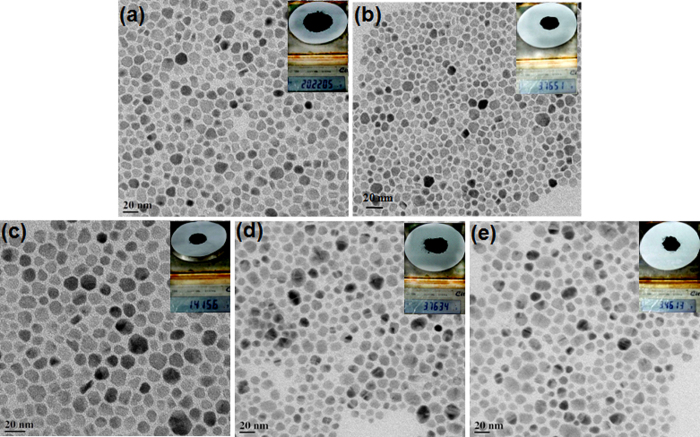
Large-area TEM image of (**a**) CZTS, (**b**) CZTSe, (**c**) CZTSSe, (**d**) CFTS, and (**e**) CMTS nanoparticles that were produced at a large scale. Inset photographs show the particular batch of nanoparticles produced at a large scale.

Transmission electron microscopy was performed to investigate the size, shape, and the distribution of the particles that were as-synthesized. Figure 3a–e shows the transmission electron microscope images of all the quaternary chalcogenide nanoparticles that were produced at a gram scale (Insets of Fig. 3). In each case, the nanoparticles are spherical, and the standard deviation of particle size distribution is σ ≤ 20%. The higher content of amine supports diffusion-controlled growth (due to low concentration of growth species), which assists in the formation of uniform small-sized nanoparticles^44^.

**Figure 4:**
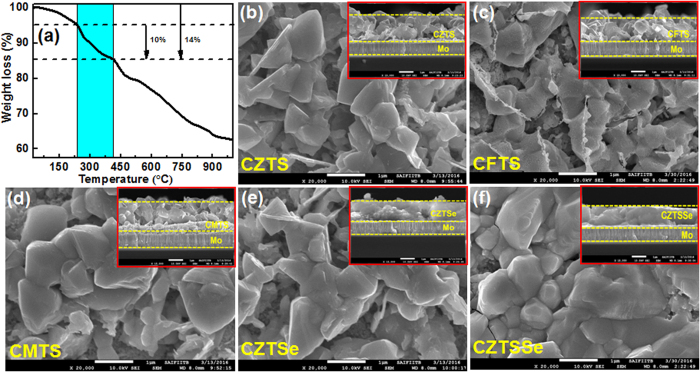
(**a**) TGA curve of the as-synthesized CZTS nanoparticles. TGA indicates that the content of the oleylamine ligands on the CZTS nanoparticles’ surfaces is about 10 wt%. SEM surface morphologies of the sulfurized/selenized (**b**) CZTS, (**c**) CFTS, (**d**) CMTS, (**e**) CZTSe, and (**f**) CZTSSe nanoparticles film on the Mo/glass substrate. The inset shows the corresponding SEM cross sections of the sulfurized/selenized nanoparticle film on the Mo-coated glass substrate.

Thermo gravimetric analysis (TGA) of CZTS samples performed under N_2_ atmosphere up to 1000 °C reveals the presence of a significant proportion of volatile compounds (around 10 wt%) in the CZTS particles (Fig. 4a). TGA curve of the CZTS nanoparticles shows a well-defined weight loss profile over a temperature range of 240–415 °C, which is attributed to the decomposition of the organic ligands that are attached to the particle surface, such as oleylamine and chemisorbed amine-modified complexes. The early weight loss of approximately 4% can be ascribed to the removal of physically adsorbed water molecules and trace solvent molecules^45^,^46^.

In addition to the films’ composition, surface morphology and cross-sectional analysis are also crucial to evaluate the uniformity and the grain size of the film. A large grain size with minimal defects and large-area uniformity are necessary for the improvement of the performance of the solar cell. Figures S5 and S6 show the SEM surface and cross-sectional SEM images of all the as-deposited films. The films show smaller grains, which is not desirable if the solar cells are to be high in efficiency. Hence the as-deposited thin films should be annealed at higher temperatures in order to achieve appropriate grain size and crystallinity. Figure 4b–f shows the SEM images of surface and cross-sectional morphologies of all the samples after sulfurization/selenization. The surface morphologies of the samples show dense and large grains as a result of the post sulfurization/selenization process. The same trend was found in the grain sizes of all the samples. The highest grain size with densely packed grains was observed in case of the CZTSe and CZTSSe, while slightly smaller grains are seen in the CZTS, CMTS, and CFTS samples. The enhanced grain growth is due to selenium inclusion in CZTS; this has also been found in most investigations on Se substitution in CZTS^38^. The increased grain size of the CZTSSe absorber layer decreases the charge carrier recombination at the grain boundaries^47^. The insets of Fig. 4 show the cross-sectional SEM images of the nanoparticle thin films after sulfurization/selenization, with thicknesses varying from 800–900 nm. All films are uniform and show a compact arrangement of nanoparticles without any voids and other structural defects such as cracks.

The compositions of all the as-synthesized, annealed and sulfurized nanoparticle films were determined using energy-dispersive X-ray spectroscopy (EDS), inductively coupled plasma atomic emission spectroscopy (ICP-AES) and are listed in Table 1. The as-synthesized CZTS, CZTSSe, and CZTSe samples are slightly rich in Zn-content and the ratios of Sn and Cu are almost stoichiometric. Annealing was carried out in the presence of argon gas to avoid Sn–S loss. From the kinetic gas theory (Hertz–Knudsen equation), maximum evaporation rate per area *A* in the vacuum can be expressed as^48^:


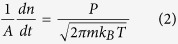


where *m* is the mass of the evaporant particles; *P* is the equilibrium vapor pressure of the evaporating particles at the temperature, *T*; and *k*_B_ is the Boltzman constant. This Hertz–Knudsen equation is valid if the evaporation process is carried out in a vacuum; however, in a gas atmosphere, this approach is not applicable any more^48^. In a gas atmosphere, there are collisions between evaporating particles and the surrounding gas particles and, thus, the evaporation is limited by diffusion and convection in the surrounding gas phase^48^. Hence, the evaporation rate in an inert gas atmosphere is always lower than the vacuum. Even though there might be apprehension about Sn–S loss after heat treatment in vacuum; there is no significant loss of Sn–S in the films after heat treatment in presence of an argon gas atmosphere.

**Figure 5:**
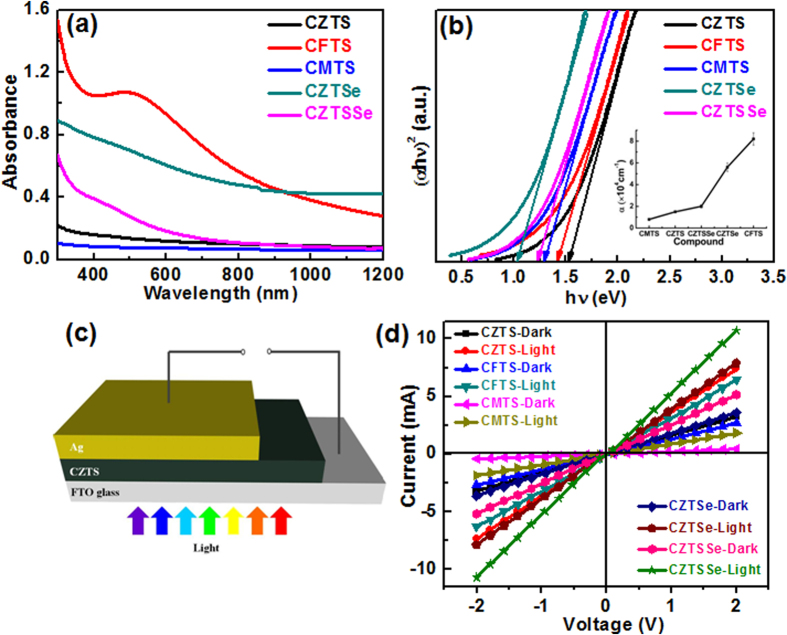
(**a**) Optical-absorption spectra of all the nanoparticles as labeled on the figure, (**b**) Tauc’s plot of various quaternary chalcogenide nanoparticles. The inset shows the absorption coefficient variations plot of various quaternary chalcogenide nanoparticles, which was calculated using Eq. (4). The substitution of different anion/cations in CZTS leads to a change in the band gap energy, from 1.5 eV to 1.04 eV, with high optical-absorption coefficients (~10^4 ^cm^−1^) in the visible region, (**c**) Schematic of the device geometry that was employed in order to study the photo response, and (**d**) I–V characteristics of CZTS, CFTS, CMTS, CZTSe, and CZTSSe thin films under an AM 1.5G simulated solar illumination.

An optimal direct band gap and high absorption coefficient is among the most basic requirements for a high performance solar cell. We characterize the UV-visible absorption spectra of quaternary chalcogenide nanoparticles (dispersed in toluene) using UV-visible spectrometer in the wavelength range of 300–1200 nm (Fig. 5a). The optical band gap of the Cu_2_MSnS_4_ (M = Zn, Fe, Mn) nanoparticles is calculated using Tauc’s plot (Fig. 5b). Figure 5a shows the absorption spectra of Cu_2_ZnSnS_4_, Cu_2_FeSnS_4_, Cu_2_MnSnS_4,_ Cu_2_ZnSnSe_4_, Cu_2_ZnSn(S_0.5_Se_0.5_)_4_ and the corresponding band gap of 1.51, 1.46, 1.28, 1.04, and 1.24 eV, respectively. These band gap values are similar to those reported in the literature^23^,^32^,^49^,^50^,^51^. The estimated band gap of CZTSSe is 1.24 eV, which is larger than that of CZTSe and less than that of CZTS. The band gap estimation by using Tauc’s plot for CZTSe family is usually not reliable due to the presence of mid gap states and the band gap strongly depends upon the composition of the films^51^,^52^. According to reference^51^, the band gap of CZTSSe can be estimated by the following equation:


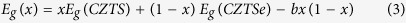


where *x* is the composition ratio of sulfur and selenium, and *b* is the specific compositional independent optical bowing constant that describes the degree of nonlinearity in the band gap. According to theoretical calculations, the *b* value for the CZTSSe sample is 0.1 eV^52^. Thus, the calculated band gap is 1.25 eV, which is very close to the experimental value. As-prepared nanoparticles show absorption in the whole visible range. In addition, CFTS spectra show a broad band that extends from approximately 400 nm to 600 nm. The absorbance band centred at 502 nm represents the excitonic band of CFTS^24^. The absorption coefficients of all the thin film samples were calculated from the UV-visible transmittance data at 600 nm (inset of Fig. 5b).

The optical-absorption coefficient (α) was calculated from the transmittance data using the following equation^53^:


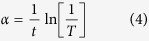


where ‘*t*’ is the film thickness, and *T* is the transmittance. Almost all samples show an absorption coefficient of an order (≈10^4 ^cm^−1^) in the visible region, which is similar to theoretical estimation and plenty of experimental observations^53^.

In order to investigate the potential of the quaternary chalcogenide nanoparticles as an active layer in photovoltaic applications, we have studied the photo-conductivity of the nanoparticle thin films. In order to measure the photo response, we used FTO as the back contact and silver (Ag) as the top contact (Fig. 5c). Current-voltage (I–V) curves of the thin films were measured, both in the dark and under illumination using a solar simulator (AM 1.5G, 100 mW/cm^2^). A current increment (by an order of 32%) is observed under illumination for all the samples, as shown in Fig. 5d. The higher current shows an effective charge transfer characteristic, which confirms that the capping agent was removed from the nanoparticles surface^54^. The dark current for samples varies in the sequence CZTSSe > CZTSe > CZTS > CMTS > CFTS thin films. Conventionally, illumination excites the electrons from the valence band to the conduction band and thus increases the hole concentration in the valence band, which enhances the conductivity of the film. This feature demonstrates the generation of photo current and confirms the potential use of these nanoparticles in photovoltaic applications.

Hall Effect measurements were carried out in order to study the electrical properties of the thin films with an applied magnetic field of 0.50 T. As calculated from the Hall coefficient, all samples exhibit p-type conductivity. The resistivity, carrier concentrations, and Hall mobility of various nanoparticles are summarized in Table 2. The CZTSSe sample shows higher Hall mobility (3.6 cm^2^/V–s) with a carrier concentration of 4 × 10^17 ^cm^−3^, and lower resistivity (3.6 Ω-cm) when compared to all other samples, which is consistent with the higher current observed in the I–V characteristics (Fig. 5d). The observed higher mobility of CZTSSe is due to the presence of larger grains and compact microstructure of the films, whereas CFTS and CMTS show lower mobility. Larger grains have fewer grain boundaries, leading to lesser scattering of the charge carriers; this reduces the recombination rate of photo-generated charge carriers. The preliminary results based on optical-absorption, photo-response, and Hall measurements demonstrate that all these nanoparticles have the potential for use as an absorber layer in thin film solar cells.

**Figure 6:**
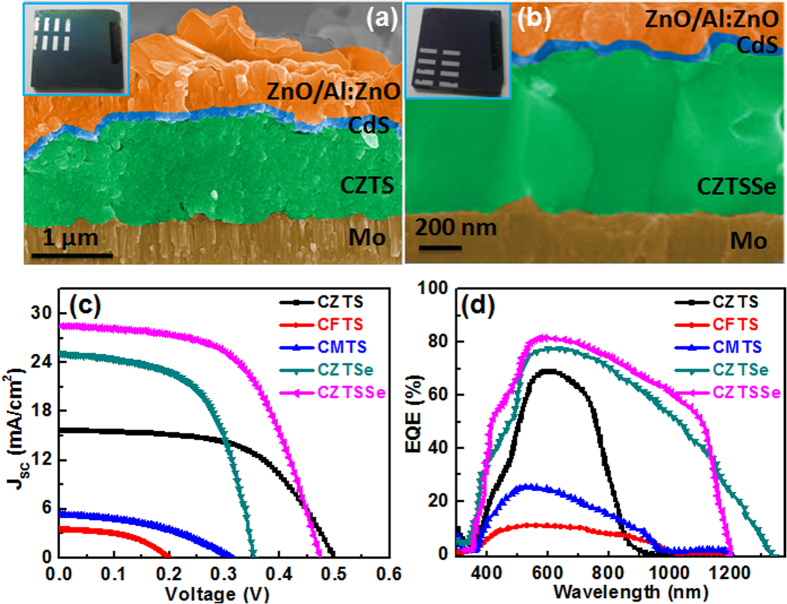
SEM cross-sectional images of (**a**) CZTS and (**b**) CZTSSe thin-film solar cells. The insets show digital photographs of as-fabricated CZTS and CZTSSe solar cells, (**c**) Current density-voltage (J–V) characteristics, and (**d**) EQE of Cu_2_MSnS_4_/Se_4_ (M = Zn, Mn and Fe) nanoparticle thin-film solar cell devices that were measured under AM 1.5G-simulated solar illumination.

Figure 6a,b show the cross-sectional SEM images of the as-fabricated CZTS and CZTSSe solar cells. The two samples consist of well-faceted large-grain crystallites. The CZTSSe contains larger grains without any noticeable voids, whereas the CZTS sample shows small voids in the cross section of the device. Figure S7 shows the cross-sectional images of the as-fabricated CZTSe, CFTS, and CMTS solar cells. The CMTS and CFTS films consist of voids at the interface of Mo and the absorber layer. The J–V characteristics of the fabricated devices are presented in Fig. 6c and Table 3. The current densities of the CZTS-based solar cell exhibit a larger open circuit voltage (V_oc_ of 498 mV than CZTSe-based solar cell V_oc_ = 354 mV), which is expected because it has a higher band gap as compared to all other devices. However, due to the improved morphology and the bigger grain size, the short-circuit current, V_oc_, of a CZTSe-based solar cell is found to be being significantly better when compared to the CZTS solar cell. In comparison, the CZTSSe devices show an efficiency of 7.4%, which is the highest, as expected from the Hall and photo-response measurements. It has also been observed that Se doping in CZTS enhances the electrical conductivity, mobility and solar cell power-conversion efficiency due to better grain growth and reduction of potential barriers at grain boundaries^31^. However, the device performance of the CZTSSe absorber in this study is still much lower than that which is produced by nanoparticle inks and hydrazine-based slurries^15^. We believe that increase of the thickness of the precursor, the optimization of the annealing treatment, and the hydrazine treatment will improve the growth of larger grains, and would ultimately lead to improved performance of the CZTSSe-based solar cells. From Hall and photo-response measurements, CZTSSe shows higher mobility; low resistivity leads to higher current density with a reasonable fill factor. When compared to the rest of the devices, the CFTS devices show poor performance due to low mobility and a higher carrier concentration, which is also confirmed by Hall, the UV-vis absorption spectra, and photo-response measurements. Even though the CFTS film has higher absorption, the solar cell efficiency is low because of low carrier mobility and high carrier concentration. In order to study the wavelength-dependent photo responses of the solar cells, EQE measurements are performed for all the devices (Fig. 6d). Near the blue region (above 450 nm) of the spectrum, the EQE data shows a sudden decay in all the devices, which is due to the absorption loss of the CdS/ZnO/AZO layers^55^. From 300–350 nm, the EQE sharply increases, which is consistent with the reported literature^55^. The EQE of the CZTS solar cell shows a maximum value of 68% at 600 nm and then drops slowly from 610 nm to 745 nm. This can be ascribed to higher reflection and recombination losses due to the low carrier diffusion length of the CZTS layer^55^. In this region, when all the devices were compared, the CZTSSe device showed a stronger photo-response (≈80%). The EQE in the visible region and near the infrared region shows more potential for CZTSe and CZTSSe cells when compared to other cells. This high EQE is mainly a result of good carrier collection due to the higher carrier mobility of generated charge carriers in the absorber layer. The cut off wavelength of CZTSSe extends to near the infra red (IR) region, whereas, the CZTS solar cell showed a cut off wavelength at 900 nm. The extended cut off wavelength in CZTSSe is attributed to the lower band gap (1.24 eV) in comparison to CZTS (1.51 eV). The replacement of sulfur with selenium lowers the band gap from 1.51 eV to 1.24 eV, which increases the current density in the CZTSSe solar cell remarkably. The CMTS and CFTS show a weak photo response in the visible region, which could be due to poor carrier collection because of low carrier mobility. Moreover, the high series resistance (R_S_) might be due to charge carrier recombination at the interface of Mo and the absorber layer because of voids. The low shunt resistance (R_SH_) values can be attributed to the smaller grain size. Thus, further optimization in the thickness of the absorber layer and in the controlling of the grain size and the device fabrication process are essential in order to increase the power-conversion efficiency of the solar cell.

## Conclusions

In this work, we have synthesized Cu_2_MSnS_4_/Se_4_ (M = Zn, Mn and Fe) quaternary chalcogenide nanoparticles by the thermolysis process. The nanoparticle films have high phase purity; the absence of secondary phases is confirmed using Raman spectroscopy and XRD measurements. It has been observed that the heat treatment in the presence of an inert gas suppressed the Sn and S losses from the films. UV-vis spectroscopy data shows that the films have a high optical-absorption coefficient (10^4 ^cm^−1^) in the visible region, and that their optical band gap varies from 1.04 to 1.51 eV. Among all the synthesized nanoparticles, highest photo current is obtained for the CZTSSe nanoparticles. We also fabricated the solar cells with these nanoparticles and tested their device performance. The efficiency of the CZTS solar cell enhances significantly, from 4.5% to 7.4%, with selenium doping. The variation of device performance may be ascribed to the change in the micro structure and the band gap. In conclusion, the observed changes in structural, optical, and device performances due to anion/cation substitution in CZTS should be further explored for obtaining higher power-conversion efficiencies in future.

## Additional Information

**How to cite this article**: Ananthoju, B. *et al*. Cation/Anion Substitution in Cu_2_ZnSnS_4_ for Improved Photovoltaic Performance. *Sci. Rep.* **6**, 35369; doi: 10.1038/srep35369 (2016).

## Supplementary Material

Supplementary Information

## Figures and Tables

**Table 1:** Compositional analysis and lattice parameters of all the nanoparticles.

Compound	Method	Precursor Ratio			Lattice parameters (Å)
		Before annealing	After annealing	After sulfurization	
CZTS	ICP	1.9:1.1:1.1:3.9	1.9:1.0:0.8:3.1	1.7:1.1:1:4.0	a = 5.42, c = 10.84
	EDX	1.8:1.2:1.0:4	1.9:1.1:0.9:3.4	1.9:1.0:1:4.2	
CZTSSe	ICP	2.1:1.0:1.0:2.3:2.4	1.9:1.0:1.1:2.3:2.2	1.9:1.0:0.9:2.1:2.4	a = 5.48, c = 11.54
	EDX	2.0:1.1:1.1:2.5:2.5	2.0:1.1:1.0:2.2:2.3	2.0:1.1:1.0:2.3:2.5	
CZTSe	ICP	1.8:1.1:1.2:3.5	2.0:1.0:0.8:2.8	2.0:1.0:1.0:4.3	a = 5.65, c = 11.56
	EDX	1.9:1.2:1.1:3.2	1.9:1.1:0.9:3.0	1.9:1.0:1.0:4.5	
CMTS	ICP	2.0:1.1:1.0:4.1	2.0:1.0:0.9:3.4	2.0:1.0:0.9:4.1	a = 3.81, c = 6.32
	EDX	2.0:1.2:1.1:4.0	1.9:1.1:0.9:3.6	1.9:1.0:1.0:4.3	
CFTS	ICP	1.8:1.1:1.0:4.1	1.9:1.1:0.8:3.9	2.0:1.0:1.0:4.0	a = 3.81, c = 6.32
	EDX	2.0:1.2:1.1:4.0	1.9:1.2:0.9:3.8	2.0:1.1:1.0:4.1	

**Table 2:** Electrical properties of nanoparticle films determined using the Hall measurement system.

Sample	Resistivity (Ω-cm)	Carrier concentration (cm^−3^)	Hall mobility (cm^2^/V-s)
CFTS	14	1.1 × 10^18^	0.09
CMTS	20	2.5 × 10^14^	0.11
CZTS	11	1.1 × 10^16^	2.6
CZTSe	7.4	3.1 × 10^17^	3.1
CZTSSe	3.6	4.0 × 10^17^	3.8

**Table 3:** Quaternary chalcogenide nanoparticle based solar cell electrical performance parameters.

Sample	J_SC_ (mA/cm^2^)	V_OC_ (mV)	E_g_ (eV)	FF (%)	R_S_ (Ω–cm^2^)	R_SH_ (Ω–cm^2^)	η (%)
CFTS	3.6	200	1.46	31	41	150	0.4 ± 0.04
CMTS	5.3	315	1.28	37	31	210	0.7 ± 0.02
CZTS	15.6	498	1.51	56	8.0	700	4.5 ± 0.12
CZTSe	25.1	354	1.04	57	6.0	420	5.1 ± 0.10
CZTSSe	29	476	1.24	59	3.5	500	7.4 ± 0.12
