# Supplementary material for: Cation/Anion Substitution in Cu2ZnSnS4 for Improved Photovoltaic Performance
Source: Sci Rep. 2016 Oct 17;6:35369. doi: 10.1038/srep35369 (PMC5066312; doi:10.1038/srep35369)
Supplement: Supplementary Information [file srep35369-s1.pdf]

Supplementary Information

Cation/Anion Substitution in Cu_2ZnSnS_4 for Improved Photovoltaic Performance

Balakrishna Ananthoju[1, 2], Jeotikanta Mohapatra[3], Manoj K. Jangid[4], D. Bahadur[1, 4], N.V. Medhekar[1, 5], M. Aslam[1, 2, 6*]

[1]IITB-Monash Research Academy, IIT Bombay, Powai, Mumbai - 400076, India,

[2]Department of Physics, IIT Bombay, Powai, Mumbai- 400076, India,

[3]Centre for Research in Nanotechnology and Science (CRNTS), IIT Bombay, Powai, Mumbai - 400076, India,

[4]Department of Metallurgical Engineering and Materials Science, IIT Bombay, Powai, - Mumbai 400076, India,

[5]Department of Materials Engineering, Monash University, Clayton, VIC 3800, Australia,

[6]National Centre for photovoltaic Research and Education, IIT Bombay, Powai, Mumbai- 400076, India

*Corresponding author:

Prof. Mohammed Aslam
Department of Physics
Indian Institute of Technology Bombay
Powai, Mumbai-400076, India
Ph: +91-22-25767585, Fax: +91-22-25767552
Email: m.aslam@iitb.ac.in

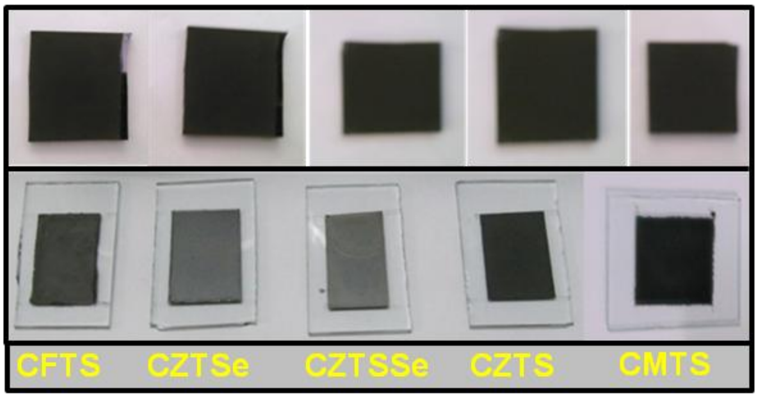

Fig. S1 Photographs of as–deposited (on glass) and sulfurized/selenized thin films (on Mo coated glass) of all the samples.

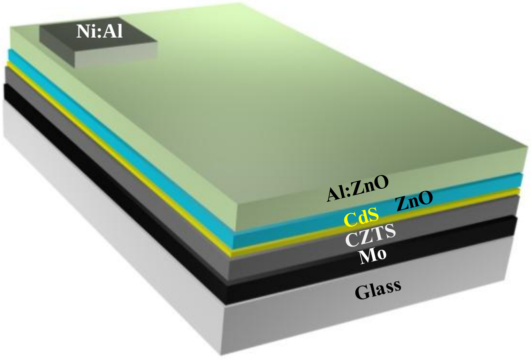

Fig. S2 Schematic cartoon design of a typical CZTS solar cell device structure. Diagram shows various layers of a CZTS thin film solar cell. Mo and Ni/Al are metal contact pads.

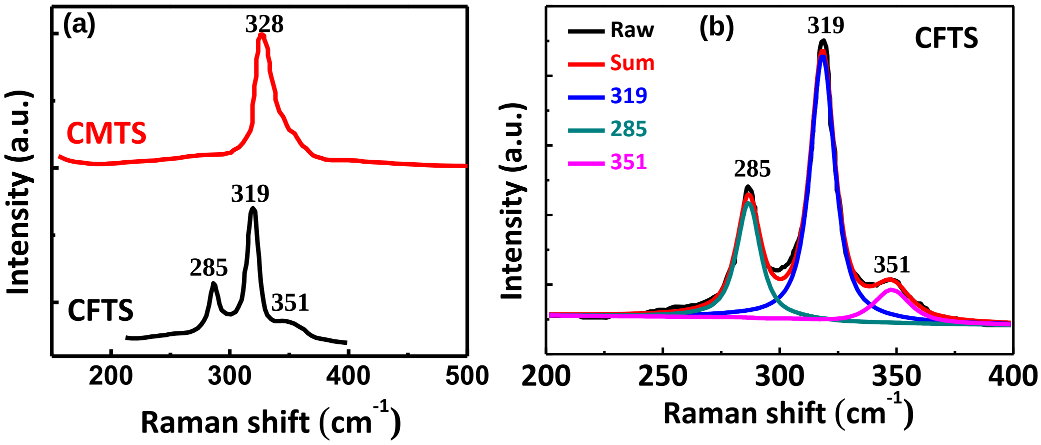

Fig. S3 (a) Raman spectra of sulfurized CFTS and CMTS nanoparticles thin film samples, (b) fitting result for the CFTS thin film.

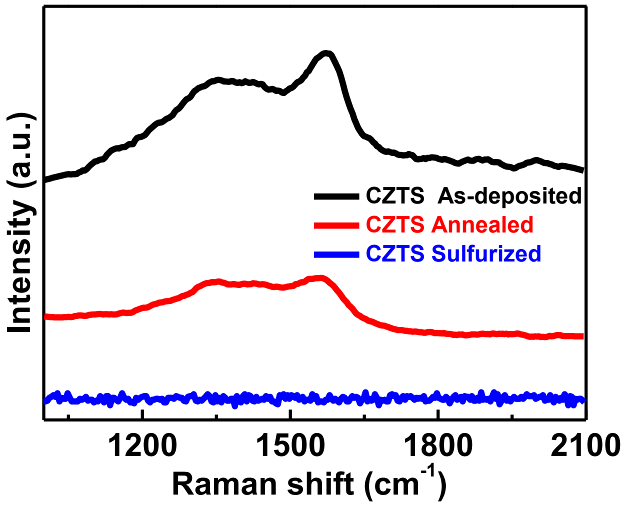

Fig. S4 Raman spectra of the CZTS as-deposited, annealed, and sulfurized thin films.

Fig. S5a shows the CZTS nanoparticle ink. Fig. S5b–f shows the SEM surface morphology images of the as deposited nanoparticle thin films. All films are uniform and show a compact arrangement of nanoparticles without any pinholes and other structural defects such as cracks. Fig. S5a-e shows the SEM cross-sectional images of the nanoparticle thin films after annealing at 400 °C with the thicknesses seen varying from 500–800 nm. All films are uniform and show compact arrangement of nanoparticles without any voids and other structural defects.

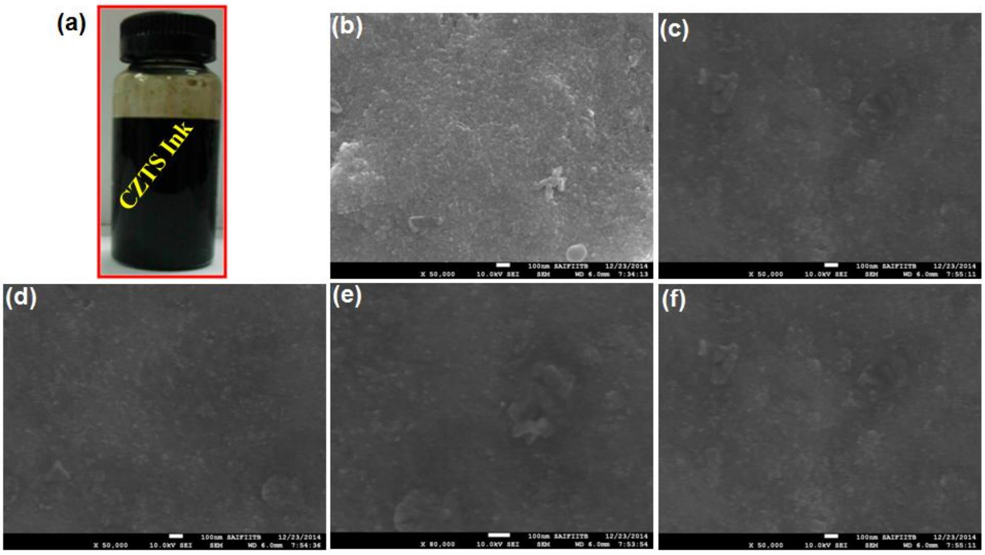

Fig. S5 (a) CZTS nanoparticles ink. SEM surface morphologies of as–deposited (b) CZTS, (c) CFTS, (d) CMTS, (e) CZTSe and (f) CZTSSe nanoparticles film on the glass substrate.

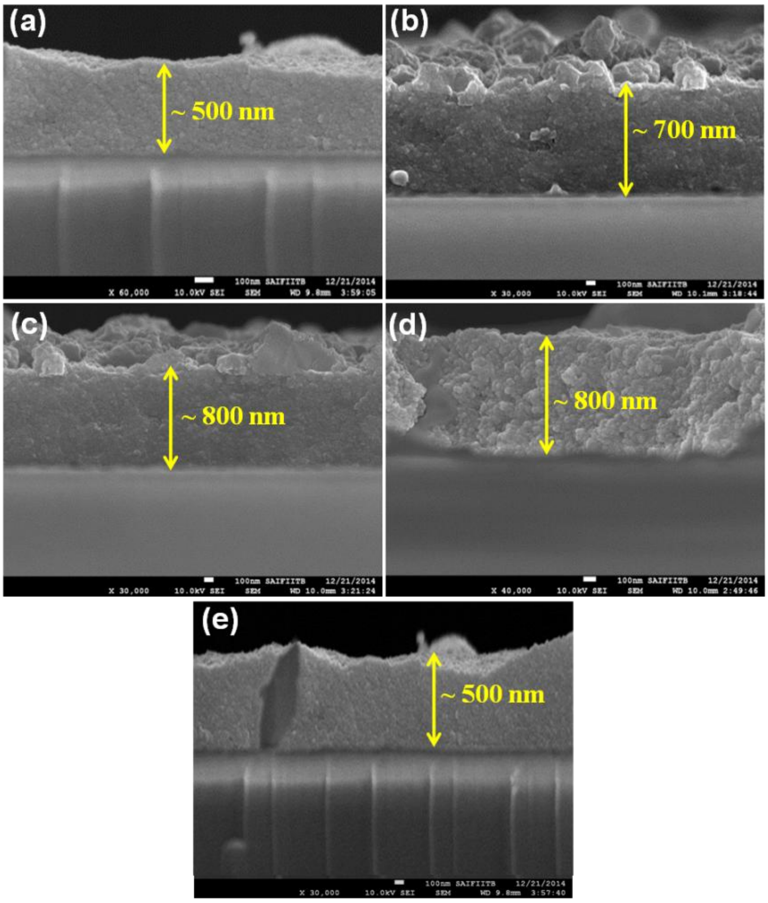

Fig. S6 SEM cross sectional images of the as-deposited (a) CZTS, (b) CFTS, (c) CMTS, (d) CZTSe and (e) CZTSSe nanoparticles film on the glass substrate.

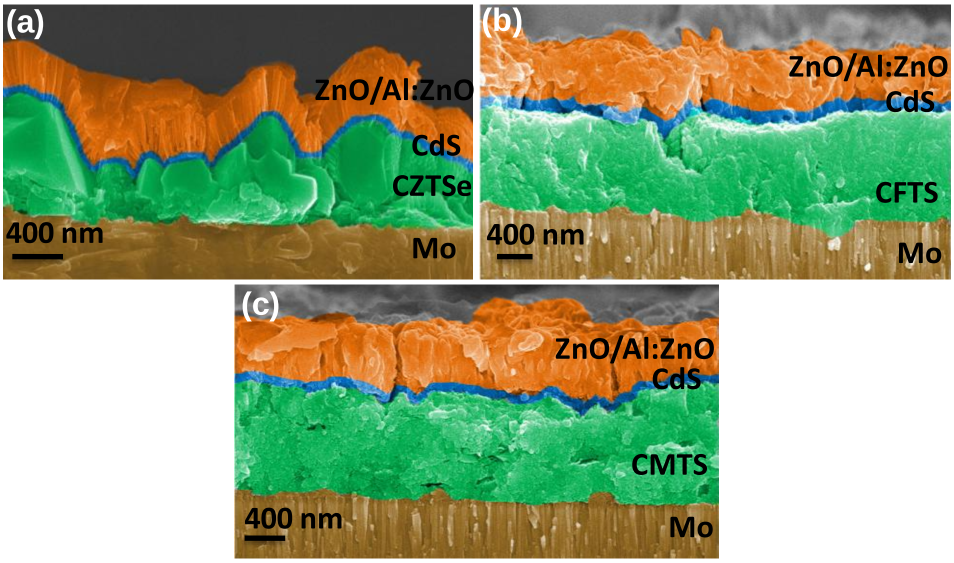

Fig. S7 SEM cross-sectional images of the as fabricated (a) CZTSe, (b) CFTS, and (c) CMTS solar cells.

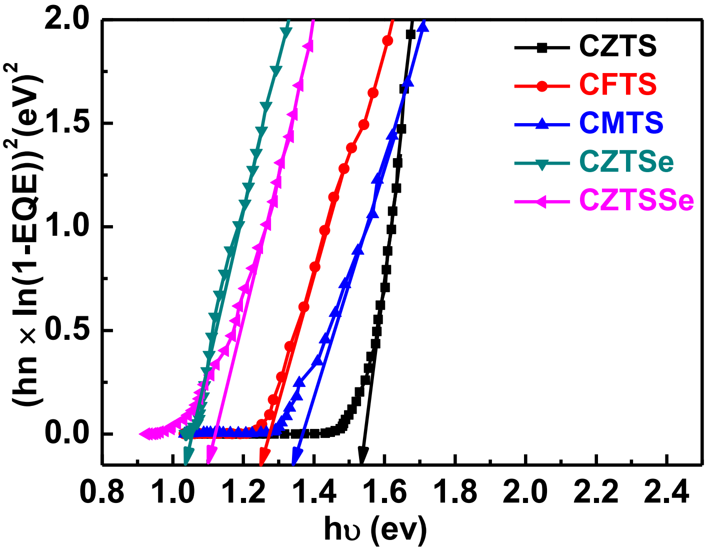

Fig. S8 The band gap of the various quaternary chalcogenide nanoparticle absorber layers obtained from a plot of $[h\nu \times \ln(1 - EQE)]^2$ vs. hν.

The band gap of Cu_2MSnS_4/Se_4 (M= Zn, Fe, Mn) nanoparticles is calculated from the plot of $[h\nu \times \ln(1 - EQE)]^2$ vs. hν, where hν is the photon energy[1]. As shown in the Fig. S8, these determined band gap values for the Cu_2ZnSnS_4, Cu_2FeSnS_4, Cu_2MnSnS_4, $Cu_2ZnSnSe_4$, $Cu_2ZnSn(S_{0.5}Se_{0.5})_4$ are 1.53, 1.26, 1.32, 1.03, and 1.12 eV, respectively, which are in reasonable agreement with the band gap extracted from the absorption spectra measurement (Fig. 5).

References:

1. K. Woo, Y. Kim, W. Yang, K. Kim, I. Kim, Y. Oh, J.Y. Kim, J. Moon, Band-gap-graded $Cu_2ZnSn(S_{1-x},Se_x)_4$ solar cells fabricated by an ethanol-based, particulate precursor ink route. *Sci. Rep*. **3**, 3069 (1–7) (2013).
